# The Role and Mechanism of Epithelial-to-Mesenchymal Transition in Prostate Cancer Progression

**DOI:** 10.3390/ijms18102079

**Published:** 2017-09-30

**Authors:** U-Ging Lo, Cheng-Fan Lee, Ming-Shyue Lee, Jer-Tsong Hsieh

**Affiliations:** 1 Department of Urology, University of Texas Southwestern Medical Center, Dallas, TX 75390, USA; U-Ging.Lo@utsouthwestern.edu (U.-G.L.); Cheng-Fan.Lee@utsouthwestern.edu (C.-F.L.); 2 Department of Biochemistry and Molecular Biology, College of Medicine, National Taiwan University, Taipei 10617, Taiwan; mslee2006@ntu.edu.tw

**Keywords:** epithelial-to-mesenchymal transition, metastasis, prostate cancer progression

## Abstract

In prostate cancer (PCa), similar to many other cancers, distant organ metastasis symbolizes the beginning of the end disease, which eventually leads to cancer death. Many mechanisms have been identified in this process that can be rationalized into targeted therapy. Among them, epithelial-to-mesenchymal transition (EMT) is originally characterized as a critical step for cell trans-differentiation during embryo development and now recognized in promoting cancer cells invasiveness because of high mobility and migratory abilities of mesenchymal cells once converted from carcinoma cells. Nevertheless, the underlying pathways leading to EMT appear to be very diverse in different cancer types, which certainly represent a challenge for developing effective intervention. In this article, we have carefully reviewed the key factors involved in EMT of PCa with clinical correlation in hope to facilitate the development of new therapeutic strategy that is expected to reduce the disease mortality.

## 1. Introduction

**Figure 1:**
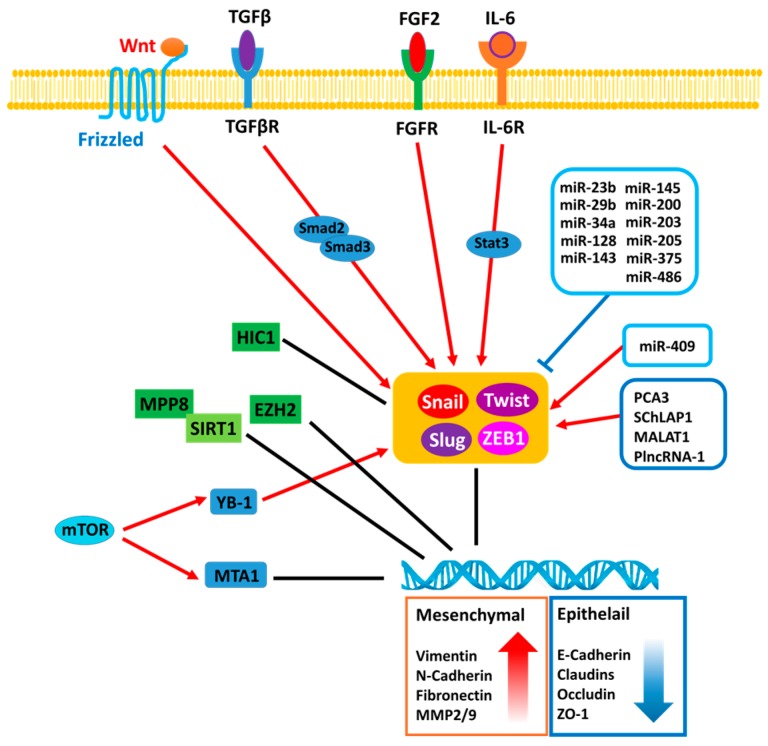
The regulatory mechanisms associated with epithelial-to-mesenchymal transition (EMT) in prostate cancer (PCa). MPP8: M-phase phosphoprotein 8; SIRT1: NAD-dependent deacetylase sirtuin-1; HIC1: Hypermethylated in cancer 1; EZH2: Enhancer of zeste homolog 2; YB-1: Y-box binding protein 1; MTA1: Metastasis Associated 1 protein; mTOR: Mammalian target of rapamycin; TGFβR: Transforming growth factor beta receptor; FGFR: Fibroblast growth factor receptor; IL-6R: Interleukin 6 receptor; Snail: Zinc finger protein SNAI1; Slug: Zinc finger protein SNAI2; ZEB1: Zinc finger E-box-binding homeobox 1; Twist: Twist-related protein 1.

The plasticity of cellular phenotypic transformation is fundamental to embryonic development. During gastrulation stage, the reprogramming process of epithelial-to-mesenchymal transition (EMT) mainly governs the phenotypic change of polarized ectodermal epithelial cells into migratory mesenchymal cells that ultimately constitute the mesodermal layer of the embryo [1]. EMT occurs by breakdown of cell-to-cell or cell-to-extracellular matrix (ECM) adherence at the polarized epithelium lining. E-cadherin is a major component of epithelial adherence junction and acts as the master gatekeeper of EMT. Loss of E-cadherin, considered to be the key step to initiate EMT, leads to collapse of intercellular mechanical communication. In contrast, critical mesenchymal markers such as vimentin and *N*-cadherin, as well as several E-cadherin transcriptional repressors including zinc finger proteins Snail/SNAI1 and Slug/SNAI2, twist-related protein 1 (Twist 1) and zinc finger E-box-binding homeobox 1 and 2 (ZEB1 and ZEB2) are highly elevated during EMT (Figure 1), leading to acquisition of mesenchymal phenotype of enhanced cell mobility [2]. EMT is critical for tissue remodeling during embryonic morphogenesis [3,4,5,6]; however, this reprogramming process is also observed in different pathological process such as organ fibrosis, wound healing and carcinoma progression. In particular, primary carcinoma cells switch from epithelial characteristics to mesenchymal-like phenotype while responding to either intrinsic genetic and molecular alteration or extrinsic microenvironmental stimuli, which leads to the invasion into surrounding stroma and subsequent vasculature, ultimately colonization at a distant pre-metastatic niche [6,7]. Particularly, the role of EMT in metastasis has been demonstrated in many cancer types including prostate cancer (PCa) to elicit their metastatic potentials [2,8,9], which is supported by significant correlation between TGF-β and EMT-related genes detected from circulating prostate cancer cells of PCa patients [10]. PCa is the most common male malignancy and the second leading cause of cancer mortality in the men of US. Current treatments for primary prostatic tumor involve radical prostatectomy, external radiotherapy, brachytherapy, and androgen deprivation therapy (ADT). The major cause of PCa mortality is the onset of metastatic castration-resistant PCa (mCRPC). Although none of these therapeutic strategies are curative for PCa, surgery and radiation remain the most effective regimen for patients with organ-confined disease. It is known that PCa is a multifocal disease with heterogeneous cell population. Thus, understanding cellular and molecular mechanisms underlying metastatic dissemination of PCa, such as EMT could generate potential therapeutic strategies to prevent PCa related mortality. In this review, we will discuss several key players in driving EMT in PCa and the different mechanisms that produce distinct signaling cascades to modulate gene transcription or epigenetic regulation, and post-transcriptional regulation by microRNA (miRNA) or long non-coding RNAs (lncRNAs).

Accumulating studies have demonstrated that activation of EMT transcription factors induces acquisition of stem cell properties in epithelial cells and contributes to the emergence of tumor-initiating cell population in several cancer types such as breast and pancreatic cancer [11,12,13]. In PCa, one study also indicates that *N*-cadherin can increase prostate tumor spheroid formation by elevating expression of stemness markers such as c-Myc, Klf4, Sox2 and Oct4 via ErbB signaling pathway [14]. In addition, the ectopic expression of Semaphorin 3 C can concurrently enhance the invasiveness and stemness in normal prostate epithelial cells, and that mesenchymal markers such as *N*-cadherin and Vimentin are highly upregulated in CD44-positive populations, compared to CD44-negative ones [15]. Using transgenic mouse model, prostate tumor cells with mesenchymal characteristics displayed enhanced invasiveness and stemness [16]. However, using primary prostate cancer-derived cells, there is no significant correlation between stemness and the expression level of EMT markers such as vimentin and *N*-cadherin [17], suggesting that more studies are needed to delineate the regulation of EMT leading to PCa stemness.

EMT-related transcription factors such as Snail, Slug and Twist are shown to confer chemo-resistance in ovarian, breast and nasopharyngeal carcinoma [18,19,20]. In PCa, a study demonstrated that reintroduction of E-cadherin significantly sensitizes chemo-resistant PCa cell lines to paclitaxel [21]. A recent study showed that Skp2-mediated Twist stabilization can facilitate the acquisition of chemo-resistant to paclitaxel or doxorubicin during PCa progression toward CRPC [22]. In addition, ZEB1 has been shown to promote the chemo-resistance in Paclitaxel-resistant PCa [23]. Taken together, the onset of EMT can lead to PCa cells acquiring drug resistance during progression.

## 2. The Signal Pathways Leading to Epithelial-To-Mesenchymal Transition (EMT) in Prostate Cancer (PCa)

The tumor surrounding microenvironment has been shown to play an important role in eliciting EMT of carcinoma cell through paracrine/endocrine fashion. Many extracellular signals are responsible for cell–cell communication that alter PCa cell behavior through a receptor-dependent manner. Several peptide hormones such as TGF-β, IL-6, FGF and Wnt are detected in prostatic stromal cells, which are associated with cancer progression [24,25,26,27] (Table 1). Among these factors existing in the tumor microenvironment, TGF-β is one of the most well characterized EMT inducer in PCa. It is known that TGF-β can promote EMT via induction of vimentin, fibronectin and suppression of E-cadherin level in vitro [28,29]. It appears that the canonical pathway of TGF-β plays a key role in increasing the expression of EMT transcriptional factor such as (Snail1/2 or ZEB1), which is supported by the evidence that E74-like factor (Elf5), a member of the large E-twenty-six (ETS) transcription factor family, can directly bind to Smad3 and block EMT [30]. Noticeably, the expression of this protein is associated with and E-cadherin expression in PCa specimens [30]. In addition to the canonical pathway, TGF-β-induced Twist expression appears to be mediated by non-canonical pathway of Stat3 in PCa cell lines such as PC3 and DU145 [31,32].

IL-6 is also demonstrated to be an EMT inducer leading to PCa invasiveness [33] and its elevated level is found in metastatic specimens of PCa patients [34]. A study investigating TRAMP (transgenic adenocarcinoma of the mouse prostate) mouse-derived PCa cell lines demonstrated elevated IL-6 levels in hormone resistant cells. Knocking down IL-6 can increase E-cadherin expression and decrease vimentin expression via Stat3 pathway, which also reduces tumor invasion in vivo [35]. Consistently, in LNCaP cells, IL-6 can induce cell migration as well as altered mesenchymal morphology. Mechanistically, IL-6 can induce Twist expression via State3 pathway and lead to the increased fibronectin expression and the inhibited E-cadherin expression [36]. In addition, a similar effect of IL-6 on EMT has been reported using BPH cells [37].

Accumulating evidence has demonstrated that fibroblast growth factor (FGF) family is associated with EMT in PCa. An in vitro study using PCa cell lines demonstrated that FGF2 increases mesenchymal markers, *N*-cadherin, vimentin and decreases epithelial marker, E-cadherin, leading to cell invasion [38]. Moreover, by using a transgenic mouse-expressing FGF9 in PrECs crossed with the TRAMP mouse model, the authors found that forced expression of FGF9 can accelerate the PCa progression in TRAMP mice. Mechanistically, FGF9 derived from LNCaP cells is shown to activate c-Jun dependent TGF-β secretion from prostatic stromal cells, which in turns triggers EMT of LNCaP cells in a paracrine manner [39].

PCa is a typical androgen-dependent disease and ADT is a standard treatment for patients with metastatic disease. It is known that androgen receptor (AR) can induce the expression of several proteases such as MMP2/9 and TMPRSS2 underlying cell invasion [40,41,42]. In contrast, AR ablation has been shown to induce EMT genes [43]. Mechanistically, AR directly represses Snail gene expression by directly binding to its responsive elements of the promoter. Thus, enzalutamide can promote PCa EMT by de-repressing Snail [44]. Similarly, AR-negative PCa cell lines, PC3 and DU145, show higher mesenchymal gene expression and lower epithelial characteristics than androgen-dependent PCa cell lines, LNCaP [45]. By using patient-derived xenograft model, a study demonstrated that both *N*-cadherin and vimentin become elevated after ADT [46]. Furthermore, ADT can affect EMT gene expression partly due to the emergence of AR variants [47,48]. For example, one of AR variant such as AR-V7 has been shown to induce mesenchymal genes such as ZEB1 and vimentin, and stem cell marker, Nanog, leading to metastasis [49,50,51].

Clinically, there is a positive correlation between AR and β-catenin in high-grade PCa [52]. It certainly implies Wnt/β-catenin signaling is associated to PCa [53]. Indeed, DAB2IP, a potent tumor suppressor, is able to block Wnt-induced EMT by facilitating β-catenin degradation resulted in increasing E-cadherin expression through the canonical pathway [54]. It has been reported that up-regulation of Frizzled 8 (FZD8), which is a subtype of Wnt family receptor, induces PCa to metastasize to bone [55]. Moreover, osteoblast derived Wnt-induced secreted protein-1 (WISP1) facilitated PC3 and DU145 invasion through up-regulation of VCAM-1 [56]. In addition, non-canonical Wnt signaling could also contribute EMT, for example, in high-grade PCa specimens exhibiting elevated Wnt5A, *N*-cadherin and vimentin expression but no change in E-cadherin expression [57].

Accumulating evidence has demonstrated that interplay between signal transduction pathways in response to external stimuli is a critical mechanism to drive the development of metastatic CRPC. In particular, signaling pathways involved in the initiation of EMT often lead to suppression of E-cadherin, resulting in enhanced cell proliferation and metastasis. Particularly, the phosphoinositide 3-kinase (PI3K)-Akt signaling pathway integrates external growth factor stimulations with internal cellular processes. TGF-β can initiate EMT by dissociation of E-cadherin/catenin complexes from the actin cytoskeleton via PI3K/Akt signaling [58]. In this study, the authors demonstrated that TGF-β treatment induces PI3K activation, phosphorylation on either α- or β-catenin associated with E-cadherin localized at the actin cytoskeleton. Dissociation of phosphorylated α- or β-catenin molecules from the E-cadherin results in diminished cell–cell adhesion, as well as enhanced cell migration and invasion. Moreover, TGF-β treatment also leads to significant down-regulation of E-cadherin protein level, accompanied by dramatic change of cancer cell shape from epithelial-like to spindle-like morphology. In contrast, PTEN is a PI3K-Akt signaling regulator involved in stabilization of adherent junctions via de-phosphorylation of β-catenin. TGF-β treatment causes PTEN dissociation from β-catenin, and thus reduced β-catenin dephosphorylation further facilitates PI3K-induced β- and α-catenin phosphorylation, leading to reduction of E-cadherin/catenin complex at the adherent junction and transformation into a more mesenchymal-like phenotype. Meanwhile, bone morphogenetic protein-7 (BMP-7) is shown to induce activation of PI3K and ERK signaling and contributes to the morphological conversion of bone metastatic PCa cell line in both 2D monolayer and 3D spheroid culture system [59]. In particular, the authors observed a significant down-regulation of E-cadherin, accompanied by up-regulation of both Twist and Slug in 3D-cultured PC3 spheroids after exposure to BMP7 treatment. In contrast, inhibition of both Akt and ERK signaling cascades abolishes BMP7-mediated EMT in PC3 cells by diminishing cell migration motility. Overall, this study suggests that initiation of EMT by BMP7 can be regulated through PI3K and ERK signaling in PCa.

Moreover, another critical downstream effector of Akt during prostate tumorigenesis is the mammalian target of rapamycin (mTOR) kinase. Hyper-activation of mTOR is observed in nearly 100% of advanced PCa [60,61]. In a study using ribosomal profiling approach, mTOR signaling mediates the translation of a specific repertoire of PCa genes involved in cell proliferation, metabolism and invasion. Based on the profiling outcome, mTOR translationally regulates genes including YB-1 (Y-box binding protein 1), vimentin, and MTA1 (metastasis associated 1) that are mainly involved in PCa invasion and metastasis [62]. Notably, ectopic expression of YB-1 can enhance translational activation of Snail and Twist, leading to down-regulation of E-cadherin and enhanced cell migration motility. In contrast, loss of YB-1 results in significant reduction of several mesenchymal factors such as Twist, *N*-cadherin and Snail [63]. Overall, this study demonstrated a critical impact of YB-1 on EMT, MTA1 is a chromatin remodeler playing an important role in PCa invasiveness. A study using MTA1 transgenic knock-in mouse model displayed an inverse correlation between MTA1 and E-cadherin level in the murine prostate tissue, and that MTA1 is shown to suppress E-cadherin expression at post-transcriptional level [64]. Meanwhile, another study also showed that MTA1 impacts on the invasiveness of PCa cells through regulating E-cadherin expression [65]. Functionally, ectopic expression of MTA1 leads to increased invasive capacity of untransformed prostate epithelial cell line [64,66,67]. In addition, studies using clinical specimen and tumor model also demonstrated a significant elevation of MTA1 in highly aggressive PCa, and that loss of MTA1 diminishes PCa invasion, bone metastasis and angiogenesis [68,69,70]. Overall, these studies demonstrated a significant role of MTA1 and YB-1 in the EMT of PCa leading to metastatic progression of the disease. In addition, different protein components of the mTOR complex such as mTORC1 and mTORC2 exhibit its significant impact on PCa metastasis. Loss of the mTORC1 or mTORC2 complex components, Raptor or Rictor, leads to attenuation of PCa migration and invasion due to elevated E-cadherin and β-catenin expression and reduced mesenchymal markers such as *N*-cadherin and vimentin [71]. Moreover, it appears that paclitaxel-resistant PCa cells are invasive [21]; this is initiated by EMT via Notch-1 signaling and suppression of E-cadherin expression. Overall, this study implies that Notch-1 signaling facilitates the mesenchymal phenotype associated with the acquisition of chemo-resistance in PCa cells.

## 3. Transcription Factors Associated with EMT

E-cadherin is an essential cell–cell adhesion molecule involved in maintaining the epithelial integrity of the carcinoma cell. Hence, loss of E-cadherin becomes a critical step for EMT initiation and is mainly regulated by several transcriptional repressors such as Snail [72], Slug [73], Twist [74,75] and ZEB1 [76].

Snail is a zinc-finger protein binding to E-box sequences of the E-cadherin promoter [72]. Aberrant up-regulation of Snail has been observed in many malignancies such as breast cancer, ovarian cancer [77,78], colorectal cancer [79,80] and PCa [44,81]. In particular, elevation of Snail protein expression has been seen in both enzalutamide-resistant PCa cell line as well as highly metastatic PCa patient specimens [81]. Particularly, ectopic expression of Snail results in enhanced elevation of both AR and AR variants, which might be an initial cause of enzalutamide resistance in PCa lines, suggesting the impact of Snail on the recurrence of metastatic CRPC. In addition to its suppressor function, Snail may facilitate cancer metastasis via enhancing the protein expression and enzymatic activity of urokinase-type plasminogen activator (uPA) leading to enhanced motility in PCa cell lines [82]. In addition, Snail impacts on the expression level of several tight junction protein components by repressing the promoter activity of claudins and occludin genes, and inhibiting Zona occludin 1 (ZO-1) expression at post-transcriptional level [83,84]. Overall, the impact of Snail on EMT process and cell adhesion molecules demonstrates its crucial regulatory role in the disease progression of PCa.

Slug is a dominant regulator of EMT in many cancers including PCa. In addition to acting as an E-cadherin transcriptional repressor, Slug also regulates other factors leading to EMT in PCa. An in vitro study showed that Slug up-regulates both CXCL12 and CXCR4 and impacts on CXCL12/CXCR4 signaling downstream target gene, MMP9, leading to highly invasiveness of PCa [85], implying that up-regulation of autocrine CXCL12 is a critical mechanism underlying Slug-mediated migration and invasion of PCa. In primary PCa, Slug/SNAI2 gene expression are often down regulated due to the promoter methylation; the expression of Slug is restored or elevated in the invasion front of high grade PCa and lymph node metastases [86]. In addition, Slug can suppress several metastasis-suppressor genes such as KISS1. Particularly, KISS1 is able to inhibit EMT by via suppressing *N*-cadherin and vimentin, and increasing E-cadherin expression then diminish tumor cell migration and invasion motility [87]. Clinically, loss of KISS1 is widely observed in primary and metastatic PCa compared with benign tissue. Restoring KISS1 expression in highly metastatic PCa cell lines results in diminishing cell invasion motility [88]. Taken together, Slug is a highly potent promoter for PCa metastasis via EMT induction, cytokine production and metalloprotease secretion.

Twist is a basic helix-loop-helix protein that plays critical roles during development and tumorigenesis. Many studies have demonstrated that Twist can activate EMT, and that it enhances cell migration via binding to the promoter of the E-cadherin gene. In the past, the mechanistic association between Twist and transcriptional repression of E-cadherin has been shown in many malignancies including esophageal squamous cell carcinoma [89], bladder cancer [90], breast cancer [91] and PCa [92]. Clinically, Twist is found highly expressed in malignant prostatic tissue when compared to BPH tissue, and its protein level is significantly correlated with Gleason grades and metastasis [75]. Meanwhile, overexpression of Twist at the marginal area of prostatic tumor has been correlated with capsule invasion and biochemical recurrence (BCR) in PCa patients receiving radical prostatectomy [93]. In addition to acting as E-cadherin repressor, Twist also facilitates EMT by regulating *N*-cadherin expression [94]. This study demonstrated that β1 integrin-mediated nuclear translocation of Twist is capable of inducing *N*-cadherin transcriptional activation via binding of Twist to the E-box regulatory element within the *N*-cadherin gene, suggesting that Twist acts as pivotal transcription factor in the metastatic progression of PCa.

ZEB1 is a zinc finger homeodomain transcriptional repressor that regulates skeletal patterning during development and suppresses E-cadherin transcriptional activity in multiple malignancies.

Clinically, ZEB1 is elevated in high-grade prostatic tumors, compared to benign or lower grade PCa specimens [95]. A recent study demonstrated that ZEB1 is physically associated with the histone H4K20-specific methyltransferase, SET8. Mechanistically, SET8-induced H4K20 methylation is implied to exert a dual function in ZEB1-regulated gene expression. Functionally, ZEB1 and SET8 cooperatively trigger EMT by suppression of E-cadherin and induction of vimentin in PCa cells, leading to the invasive potential of PCa [96]. Moreover, an in vitro study has demonstrated that elevation of ZEB1 and loss of E-cadherin is concurrently observed in a subpopulation of PC3 cells that acquired trans-endothelial migration characteristics in vitro, compared to the parental cell line. In contrast, loss of ZEB1 partially restores the epithelial phenotype and reduces trans-endothelial extravasation of PC3 cells [76]. Overall, this study suggests that ZEB1 is a critical regulator of EMT and mediates vascular extravasation of PCa cells during the disease progression.

Forkhead box (FOX) proteins constitute a large family of 19 subgroups of transcriptional regulators that contain an evolutionary conserved DNA binding domain (Forkhead or winged-helix). Among them, FOXA1 is known as a pioneering transcription factor for AR [97,98,99]. However, FOXA1 loss is often detected in metastatic PCa specimen [100] because FOXA1 has an AR-independent function on suppressing EMT via regulating Slug in PCa cells. In contrast, FoxO family is able to block EMT in malignant cells of multiple cancers [101,102]. Clinically, emerging evidence has demonstrated an inverse correlation between FoxOs level and PCa grade as well as tumor dissemination, indicating its suppressor role in PCa metastasis [103]. Mechanistically, the transcriptional activity of FoxO3a is negatively regulated by PI3K/Akt signaling through post-translational phosphorylate modification. During PCa progression, progressive activation of Akt leads to increased phosphorylation of FoxO3a, which impacts on its nuclear localization and hence FoxO3a-dependent transcriptional activity is further inhibited [104]. Functionally, FoxO3a can directly compete with T-cell factor (TCF) for the interaction with β-catenin, leading to inhibition of β-catenin/TCF transcriptional activity and thus reduction in expression of β-catenin-target genes, such as ZEB1 and Snail. Moreover, knockdown of FoxO3a leads to elevation of *N*-cadherin, fibronectin, ZEB1 and vimentin in highly metastatic PC3 cells [105]. Overall, these data demonstrated a crucial role of FoxO family in PCa metastasis via targeting EMT factors.

## 4. Epigenetic Regulation of EMT

Epigenetic regulation is considered as a key initial step in mammalian development. Since EMT occurs during embryogenesis, it is conceivable that epigenetics also plays a critical role in pathologic EMT. Accumulating evidence has demonstrated that both hyper- and hypomethylation of DNA are involved in the deregulation of several genes contributing to PCa progression [106,107,108]. In particular, aberrant DNA hypermethylation in cancer may lead to inactivation of tumor suppressor genes, leading to increased invasiveness of PCa. HIC1 is a tumor suppressor gene located at 17p13.3, a chromosomal region that is frequently hyper-methylated or deleted in human tumors. HIC1 acts as a transcriptional repressor involved in the suppression of SIRT1 and the regulation of TP53-dependent apoptotic DNA-damage responses [109]. A study using PCa specimens showed that high frequency of HIC1 gene hypermethylation is observed in metastatic PCa, compared to primary and benign tissue. Moreover, hypermethylation of HIC1 gene in PCa cells leads to induction of cell migration and metastasis by promoting EMT via enhancing both Slug and CXCR4 expression that are crucial to PCa metastasis [110]. Meanwhile, restoring HIC1 expression in several PCa cell lines markedly inhibits cell proliferation, migration and invasion in vitro, as well as reduces tumor growth, tissue metastasis and bone destruction in vivo [111,112]. Clearly, epigenetic modification of HIC1 promoter can impact EMT induction in PCa.

Moreover, histone modification of critical genes has similar effect on EMT induction during PCa metastasis. The histone methyltransferase, MMSET/WHSC1 (Multiple Myeloma SET domain), is capable of facilitating EMT in PCa cells via induction of Twist1, which in turns suppresses E-cadherin expression [113]. In addition, Zeste homolog 2 (EZH2) is a critical component of Polycomb repressive complex 2 (PRC2) and causes gene silencing by increasing histone methylation. Increased level of EZH2 has been observed in PCa and many other cancer types. Particularly, transcriptional repression of E-cadherin by EZH2 is often observed in highly aggressive PCa [114,115,116]. In addition, EMT-related transcription factor can be an epigenetic regulator to orchestrate EMT process. SIRT1 is, known as class III Histone deacetylase, also characterized as an EMT-related transcription factor. By silencing of SIRT1 can cause down-regulation of ZEB1. In addition, recruitment of SIRT1 at the promoter region of E-cadherin can be facilitated by the presence of ZEB1 in PCa cells, leading to transcriptional suppression of E-cadherin [117]. A recent study demonstrated that silencing of SIRT1 can suppress PCa cell migration and invasion via down-regulation of Vimentin and *N*-cadherin, leading to subsequent up-regulation of E-cadherin [118]. Overall, SIRT1 is a unique epigenetic regulator as well as EMT-related transcription factor in PCa.

## 5. MicroRNA Associated with EMT during PCa Progression

MicroRNAs (miRNAs) are small non-coding RNA molecules regulating gene expression via post-transcriptional silencing of target genes. miRNA regulation is highly associated with multiple biological processes such as differentiation, proliferation, migration, survival and invasion. Several miRNAs are known to target transcription factors contributing to the mesenchymal phenotype in PCa (Table 2). For incidence, members of the miR-200 family (miR-200a, miR-200b, miR-200c, miR-141 and miR-449) are markedly down-regulated during PCa progression and are shown to suppress EMT mainly by inhibiting E-cadherin repressors such as ZEB1 and ZEB2 at the post-transcriptional level [119,120,121]. Both miR-203 and miR-205 are known to restore epithelial phenotype in PCa cells by targeting Slug/SNAI2 and ZEB2. Clinically, expression level of miR-203 is significantly attenuated in bone metastatic PCa specimens compared with benign tissue, while miR-205 is found to be decreased dramatically in lymph node metastasis when compared to primary prostatic tumor [122,123]. Meanwhile, by miRNA microarray analysis, miR-508-5p, miR-145, miR-143, miR-33a and miR-100 were found to be significantly down-regulated in metastatic PCa compared to the primary tumor. In particular, miR-143 and miR-145 derived from the same cluster are shown to reverse EMT and reduce PCa cell migration and invasion by targeting fibronectin and ZEB2 [124,125]. Moreover, several mesenchymal factors such as *N*-cadherin, Twist and Snail are regulated by miR-29b, which is also down-regulated significantly in PCa cell lines and PCa patient specimens when compared to normal prostate epithelial cells and adjacent benign tissue, respectively. Ectopic expression of miR-29b in PCa cells is capable of suppressing PCa invasiveness in vitro, and diminishing secondary colonization at the lungs and liver following intravenous injection in vivo, suggesting miR-29b acts as an anti-metastatic miRNA that is down-regulated during PCa progression [126]. Meanwhile, miR-23b is found to be a methylation-silenced tumor suppressor that inhibits EMT via directly targeting Src kinase and Akt. Moreover, this study also demonstrated that ectopic expression of miR-23b in PC3 cells causes decline in mesenchymal markers vimentin and Snail, and increase of epithelial marker, E-cadherin [127]. Similarly, miR-34a is a tumor suppressive miRNA implicated in EMT and cancer stemness in multiple tumors. A study showed that miR-34a is negatively correlated with PCa migration and invasion by targeting lymphoid enhancer-binding factor-1 (LEF1), a key transcription factor involved in regulation of cell proliferation and invasion. This study also demonstrated that ectopic expression of miR-34a causes the down regulation of *N*-cadherin and Snail, and induction of E-cadherin in LNCaP and C4-2B cell lines, overall suggesting that miR-34a-LEF1 regulation plays an important role in the metastatic progression of PCa [128]. In addition, miR-486 is significantly down-regulated in metastatic C4-2 cells as well as disseminated tumors in PCa patients, compared to parental LNCaP cell and localized PCa tissues, respectively. Functionally, miR-486 is demonstrated to target Snail by post-transcriptional suppression and functionally inhibit PCa cell migration and invasion [129]. Findings from this study suggest that miR-486 negatively mediates the migration and invasion potential of PCa via targeting Snail.

In contrast to tumor suppressor miRNAs, aberrant expression of oncogenic miRNAs is observed in highly aggressive PCa associated with EMT. A study using intra-cardiac inoculation of PCa cells in mice demonstrated the oncogenic role of miR-409 in PCa bone metastasis [130]. Inhibition of miR-409 in highly metastatic PCa cells reverses EMT process by increasing E-Cadherin expression, reducing *N*-cadherin level, and causing morphological change to the epithelial phenotype.

In addition, several studies demonstrated a negative feedback loop between miRNA and EMT transcription factors. For example, ZEB2 known as a direct target of miR-145 can also suppress miR-145 at transcription level. This double negative feedback loop between ZEB2 and miR-145 determines the invasiveness and stemness properties of PCa and contributes to the bone metastasis [131]. Moreover, ZEB1 can suppress the transcription of miR-375 that can inhibit EMT-elicited cell migration and invasion via targeting YAP1 [132].

## 6. Long Non-Coding RNA Regulation of EMT in PCa

Long non-coding RNAs (lncRNAs), such as the prostate specific prostate cancer antigen 3 (PCA3/DD3), also plays a critical role in PCa EMT. Silencing of PCA3 in LNCaP cells modulates the expression pattern of several cancer-related genes coding EMT markers such as MTA2 and PLAUR. Meanwhile, PCA3 is shown to facilitate PRKD3-mediated invasion and migration via competitive sponging of miR-1261 [139]. In addition, SChLAP1 (Second Chromosome Locus Associated with Prostate-1) is prevalently expressed in a subset of metastatic PCa, compared to localized primary PCa. Mechanistically, SChLAP1 is able to enhance PCa metastasis by altering the cellular localization and gene regulation of tumor-suppressive SWI/SNF (Switch/Sucrose Nonfermenting) chromatin-modifying complex through interaction with SNF5 [140]. In addition, a recent study also demonstrates that SChLAP1 can modulate the MAPK1 signaling pathway, leading to accelerating cell proliferation and enhancing metastatic potential of PCa in vitro and in vivo [141]. Another highly up-regulated lncRNA in PCa is Metastasis-associated Lung Adenocarcinoma Transcript 1 (MALAT1) that is shown to enhance EZH2-mediated repression of Polycomb-dependent target gene, E-Cadherin. Mechanistically, by interacting with the Polycomb protein enhancer of EZH2, MALAT1 is capable of facilitating EZH2 recruitment to target genes, such as E-cadherin and DAB2IP, resulting in enhanced EZH2-mediated migration and invasion in aggressive CRPC cell lines [142,143,144]. PlncRNA-1 has been shown to induce *N*-cadherin expression through modulating TGF-β1 signaling, and hence increase PCa cell migration and invasion motility [145].

## 7. Conclusions

The initiation of EMT is considered the initial step leading to cancer metastasis that is expected to contribute to the poor prognosis of cancer patient. Thus, targeting EMT is likely to improve the overall survival of a patient. EMT is a highly regulated process that can be engaged by the reciprocal interaction between tumor surrounding microenvironment and cancer cells. Through extensive survey in PCa, several key inducers associated with the specific signaling pathways and their regulations have been reported. With respect to the role of EMT in cancer metastasis, stemness, and chemo-resistance, apparently, these key regulators can be druggable targets to be a new generation of cancer medicine as a targeted therapeutic strategy. In this case, small molecule inhibitor such as EZH2 inhibitor or certain unique miRNA such as miR-200 [146] and miR-145 [147] can be further tested in vivo to evaluate their efficacy and validate their mechanism of action.

## Figures and Tables

**Table 1:** Microenvironment soluble factors involved in EMT progression of PCa.

Soluble Factors	Role in EMT	Impacts on PCa Progression	Reference
TGF-β1	Inducer	Invasion, Migration, Metastasis, Sphere formation	[28,29,30,31,32]
BMP	Inducer	Sphere formation	[58]
IL-6	Inducer	Invasion, Metastasis, Sphere formation, Tumor incidence	[33,34,35,37]
FGF	Inducer	Invasion, Metastasis	[38,39]
AR	Suppressor	EMT Suppression	[24,43,44,52]
AR variants	Inducer	Metastasis	[47,48,49,50]
Wnt/β-catenin	Inducer	Invasion, Metastasis, Stemness	[42,52,53,55,56,57]

EMT: epithelial-to-mesenchymal transition; PCa: prostate cancer.

**Table 2:** MicroRNAs involved in the EMT and metastatic progression of PCa.

MicroRNAs	Role in EMT	Target	Impacts on PCa Progression	Reference
miR-200b	Suppressor	ZEB1, ZEB2	Suppress cell proliferation, EMT, invasion, and inhibit prostate tumor growth and metastasis.	[120,133,134]
miR-141	Suppressor	ZEB1, CD44, EZH2, Rac1	Inhibits cell sphere formation, invasion, and suppresses tumor regeneration and metastasis.	[119]
miR-203	Suppressor	ZEB2, Bmi, Survivin, RunX2	Suppress prostate tumor metastasis, inhibit cell proliferation, EMT, and invasion motility	[122]
miR-205	Suppressor	c-SRC, ZEB1, ZEB2	Attenuate cell proliferation, invasion and tumor growth	[123,135,136]
miR-143	Suppressor	Fibronectin, ZEB2, MMP13	Suppress cell invasion and migration	[125,137]
miR-145	Suppressor	Fibronectin, ZEB2	Repress cell bone metastasis, invasion and migration	[125]
miR-29b	Suppressor	*N*-cadherin, Twist1, Snail	Suppress cell invasion, migration and attenuate prostate tumor lung metastasis	[126]
miR-23b	Suppressor	Slug, Vimentin, Src	Suppress cell migration, invasion and attenuate prostate tumorigenecity	[127]
miR-34a	Suppressor	LEF1, *N*-cadherin, Snail	Attenuate cell invasion and migration	[128,138]
miR-486	Suppressor	Snail	Suppresses migration and invasion of cells.	[129]
miR-409	Inducer	STAG2, RBL2, RSU1, NPRL2	Increase invasiveness and aggressiveness, and promotes tumorigenecity, EMT and stemness of prostate tumor	[130]
